# Exploration on ability of printable modified papers for the application in heat sublimation transfer printing of polyester fabric

**DOI:** 10.1038/s41598-023-33546-9

**Published:** 2023-04-21

**Authors:** Abeer M. Adel, Nivin M. Ahmed, Mohamed A. Diab, Fatma N. El-Shall, Nabila El-Shinnawy

**Affiliations:** 1 Cellulose and Paper Department, National Research Centre, 33El-Bohouth St. (Former El-Tahrir St.), P.O. 12622, Dokki, Giza Egypt; 2 Polymers and Pigments Department, National Research Centre, 33 El-Bohouth St. (Former El-Tahrir St.), P.O. 12622, Dokki, Giza Egypt; 3 Dyeing, Printing and Textile Auxiliary Department, National Research Centre, 33 El-Bohouth St. (Former El-Tahrir St.), P.O. 12622, Dokki, Giza Egypt

**Keywords:** Environmental sciences, Chemistry, Materials science, Nanoscience and technology

## Abstract

In this work heat transfer papers were loaded with a new core–shell pigment based on precipitating thin shell of titanium dioxide on a core of rice husk silica ash TiO_2_/RHSA to be applied in dye sublimation printing of textile fabrics. Besides, 0.1% (w/w) cationic polyacrylamide (CPAM) and 1% (w/w) bentonite (Bt) were also added sequentially to improve drainage and filler retention of the paper hand-sheets made from bleached kraft bagasse pulps. The effect of the new core–shell pigment on the mechanical and barrier properties, thermal stability and surface morphology of modified paper sheets were investigated. In addition, the study of transfer printability and ease of dye release from paper to fabric in this heat transfer printing of polyester fabrics using silk-screen printing under different transfer parameters were studied. Also, fastness measurements including washing, light and perspiration of printing polyester fabric were also estimated.

## Introduction

Paper is a flexible material made up from cellulose pulp, which is derived from many natural sources and is used in numerous applications^1^. One of the most important uses of paper is printing, which is a method of reproducing images or texts using a template or master form on a solid surface, e.g. paper, textiles, and walls. Sublimation heat transfer printing is considered one of the most important, cleanest, and newest styles of printing^2^. This simple printing method has a number of desirable features, such as reduced need for energy and water, no need for post treatment, cheap printing technique, lower maintenance and equipment investment, and an effluent-free method in addition to producing a clear and environmentally clean image^3,4^. In spite of the many advantages of heat transfer printing, the most important drawback is that the paper that though it can be used once or sometimes twice in rare cases, and according to the paper cost, this process can be very expensive. Paper properties such as roughness, porosity, and pore size, gloss, permeability, color properties, and whiteness are the most significant factors that have an effect on printability (the consequence of interactions between printing paper, ink, and device) and the process of completing the transfer and the appearance of the image and the color degree of printers^5,6^. Furthermore, both fiber size and paper porosity play an essential role in the acceptance of the ink. Also, the existence of some filler, sizing agents or additives can provide special features to the paper.

Clay, calcium carbonate, kaolin, talc, and titanium dioxide are examples of the most used fillers^7,8^. Their usefulness is not only economically but they can also impart to the paper important properties such as opacity, bulk, smoothness, pulp dewatering and retention that are crucial for the paper quality.

Rice husk (RH) contains cellulose (50%), lignin (30%), and organic compounds (20%) with reduced nutritional value and high silica content^9,10^. Rice husk ash (RHSA) is a light, bulky, and highly porous material that is used in a variety of industries including ceramics, brick, and concrete. One of these applications is its usage as filler in paper making, but exposing the paper to both high temperatures and pressure during heat transfer printing needs strong paper that can withstand the harsh printing conditions without being torn or burned. Thermal stability, strength, and mechanical resistance are the most important characteristics that must be taken into consideration on applying a specific paper in sublimation heat transfer printing. In contrast to traditional printing paper, heat transfer printing paper should be designed to release the highest possible amount of ink or dye on its surface^11^.

Core–shell pigments are a new trend of structured pigments, containing thin shell not exceeding (15–20%) deposited on a major core representing about (85–90%) of the pigment composition. This new structured pigments with its different components can overcome the deficiencies of its individuals, besides imparting new and enhanced properties to the field of application. A good choice of both components (core and shell) can bring out-performing behavior. The beneficial use of core–shell structured pigments is based on that the shell is very thin and it is the component that is directly exposed to the surrounding medium, i.e. can undergo the main reaction with the medium and since it only covers the core partially, then the core can still have part in the reaction being not completely shielded. The structured core–shell pigment used in this work are composed of titanium dioxide shell precipitated on rice husk silica ash core^7,12,13^.

Bentonite consists mainly of montmorillonites (up to 90%) with traces of silica, quartz and its general chemical formula is Al_2_Si_4_O_10_(OH)_2_. In paper making, bentonite is used for two objectives;

- Adsorbing contaminants such as wood colloids, which interact with process additives to improve the drainage and retention.
- In addition, the interactions may occur between bentonites and cationic polymers or colloids. These interactions result from the electrostatic forces due to the bentonite high surface charge density, and they are most likely affected by the swelling extent of the bentonite and the resulting surface area^14,15^.

The use of retention aids is particularly required on the addition of mineral fillers into the pulp suspension due to their small size. Generally, adding polymers (e.g. cationic polyacrylamide), which are used as retention aids into the fibrous suspension can lead to creating positive charge, in such a way that they can be adsorbed onto the anionic particles (cellulosic fibers and fines, fillers), and thus can induce flocculation by bridging mechanisms significantly to increase the retention^16^.

This research work is designed to study the effect of new core–shell pigment based on precipitating titanium dioxide as a shell on the surface of rice husk ash representing the core. This new pigment role on the printability of bleached bagasse paper sheets and its effect on their thermal stability was studied. Also, the research was extended to study the possibility of using printed modified papers as heat transfer papers and the study of their transfer printability (ease of dye release from paper to fabrics) in heat sublimation transfer printing of polyester fabrics under different transfer conditions (times and temperatures). In addition, fastness measurements of printed polyester fabrics (washing, light, and perspiration) were estimated according to the standard methods.

## Experimental

### Materials

Rice husk (RH) was collected from the North Delta Governments of Egypt, washed, oven dried at 105 °C overnight, and ignited in a muffle at 650 °C for 3 h to form rice husk silica ash (RHSA), which is used without any further treatment. Bleached kraft bagasse pulp was supplied by Quena Company for Pulp and Paper, Quena, Egypt. Bentonite (Bt) was supplied by Egypt Bentonite & Derivatives Company. It is characterized by its low content of montmorillonite (59%), high silica content (18%). 1% (w/w) bentonite suspension was prepared by using a stirrer at 4000 rpm for 4 h. Commercial cationic polyacrylamide (CPAM) of high molecular weight (about 1.10^6^) from BASF (Percol®55) was diluted to 0.1% (w/w) by dissolving it in deionized water under stirring. Ground calcium carbonate (GCC) was supplied by OMYA Company under the trade name Hydrocarb®. Polyester fabric of 150 g/m^2^ (supplied by a company in the private sector of Egypt) was treated with 0.5 g/l anionic detergent and 1 g/l sodium carbonate at 70 °C for 1 h, and then they were washed and air-dried at room temperature. Bercolin CPK was supplied by Berssa-Turkey as a thickening agent; dispersed dye Dianix Classic Turquoise Blue S-GL (Disperse Blue 60) was supplied by Dye Star Company; and 60gm of transfer paper was supplied by Protucal Soporcel Company.

### Methods

#### Preparation of core–shell Titanium dioxide/rice husk silica ash (TiO_2_/RHSA) pigments

Rice husk was calcined at 650 °C, and then it was ground to the finest form through ball milling to form rice husk silica ash RHSA. After the formation of RHSA; 2 mg of Titanium tetrachloride were added to 100 ml hydrochloric acid, then the ground RHSA was immersed in this solution while very high rpm stirring. After the vapors were gone, the solution was left to settle down, and then 30% ammonia solution was added drop-wisely to the formed paste to aid the precipitation of titanium dioxide on the surface of RHSA and adjust the pH of the solution to assure the full precipitation of titanium dioxide on the surface of RHSA. After that, the formed paste was filtered through Buchner system and washed thoroughly. The last step was calcining the paste at 750 °C and then subjecting it to ball milling to reach a suitable particle size.

#### Instrumental characterization

##### Scanning electron microscopy (SEM)

Scanning electron microscopy (SEM) photos of the different samples were recorded on a Quanta FEG-250 microscope at a voltage of 10 kV. To avoid charging, the samples were sputter-coated with gold before scanning.

##### Transmission electron microscopy (TEM)

The prepared pigment was examined using (JEOL JX 1230) technique with micro-analyzer electron probe (Japan). This technique shows a cross-section of the particles and determines the particle sizes of the prepared pigments.

##### X-ray fluorescence (XRF)

Elemental analysis and chemical composition for RH and RHSA were carried out using energy dispersive x-ray fluorescence spectroscopy (Axios 2005, PANalytical, Netherlands).

##### Thermo-gravimetric analysis (TGA)

The TGA and DTG of modified papers were investigated by differential scanning calorimeter instrument. On the SDTQ-600 (TA-USA) thermo balance instrument, 10 mg sample was heated to 800 °C at a heating rate of 10 °C min^−1^ in a nitrogen flow at a rate of 100 ml min^−1^.

#### Preparation and characterization of paper sheets

The paper sheets were prepared using the Swedish Standard Method (SCAN-CM 64:00) and bleached kraft bagasse pulp was beaten in a valley beater until 30^o^SR. Hand sheets at the basis weight of 68 g/m^2^ were formed, and the amounts of ground calcium carbonate, GCC, TiO_2_, RHSA, and TiO_2_/rice husk silica ash (TiO_2_/RHSA) were dosage in (5, 10, 15, 20, and 25%). These fillers were added to the pulp suspension and stirred for 15 min. The CPAM was added, followed by bentonite with its optimum concentration (0.1% CPAM and 1% Bt) that is based on a previous study^17^. After each addition, the pulp suspension was stirred for one minute. Finally, the suspension was poured into the sheet formed by diluting it to about 6-7L and draining it. The hand sheets were then conditioned at 25 °C and 50% relative humidity before testing. List of abbreviation and name of samples are included in Table 1.

**Table 1:** List of abbreviations.

Name	Meaning
RH	Rice husk
RHSA	Rice husk silica ash
CPAM	Cationic polyacrylamide
Bt	Bentonite
S0	Blank bagasse paper sheet
S1	20% CaCO_3_
S2	20% TiO_2_
S3	20% RHSA
S4	20% TiO_2_/RHSA
S5	20% CaCO_3_ + 0.1% CPAM
S6	20% TiO_2_ + 0.1% CPAM
S7	20% RHSA + 0.1% CPAM
S8	20% TiO_2_/RHSA + 0.1% CPAM
S9	20% CaCO_3_ + 0.1% CPAM + 1% Bt
S10	20% TiO_2_ + 0.1% CPAM + 1% Bt
S11	20% RHSA + 0.1% CPAM + 1% Bt
S12	20% TiO_2_/RHSA + 0.1% CPAM + 1% Bt
CTP	Commercial transfer paper

##### Strength measurements

The strength properties (tensile, burst, and tear indices) of the paper sheets were determined according to Tappi standards. The average and standard deviation for the measurements were calculated based on five replicates for each sample. Tensile strength testing was measured according to the TAPPI (T494-06) standard method using a universal testing machine (LR10K; Lloyd Instruments, Fareham, UK), with a 100-N load cell at a constant crosshead speed of 2.5cm/min.

##### Estimation of filler retention

Paper sheets with the target amount of filler incorporated in each sheet (0.24 g) were incinerated in a muffle oven at 525 °C accordingto TAPPI T211om-93, and the ash weight values were calculated. Filler retention (R) was evaluated and estimated using the following equation;

$${\text{R}}\% = {\text{A}}_{1} - {\text{A}}_{2} /{\text{A}} \times 100$$

where A_1_ is the total weight of ash in the sheet loaded with filler, A_2_ is the weight of ash in the non-loaded sheet originating from pulp fibers, and A is the weight of filler included in each sheet.

##### Air permeability

The air permeability values of the paper sheets were measured using bendtsen smoothness and porosity tester made in Denmark, Andersson Sorensen, Copenhagen (air permeance tester) based on ISO 5636:3. Air permeability was determined by measuring the rate of air flow under standard pressure between the paper surface and two concentric, annular metal rings applied to the paper. The air permeability of a paper web is a physical parameter that characterizes the degree of web resistance to air flow.

##### Water absorption (Cobb test)

Water absorption of modified paper sheets loaded with (TiO_2_/RHSA) core–shell was measured. The Cobb test method is usually conducted for water absorption measurement. The samples were cut into rectangle of size 10 × 10 cm, and the weighed paper sheets were placed in a Cobb apparatus; 100 ml of water was added over the paper and left for 60 s, and then poured. The paper sheet was well dried between two filter papers and weighed.

$${\text{Cobb}}\;\left( {{\text{g/m}}^{2} } \right) = \left( {{\text{Weight of wet paper sheet}} - {\text{Weight of dry paper sheet}}} \right) \times 100$$

#### Printing paste and silkscreen printing

The printing paste recipe is set as follows; 3:3:94 (disperse dye:synthetic thickener:water). The prepared and commercial transfer paper sheets are manually printed by the previous formulation, using a silkscreen technique and left to air dry.

##### Transfer printing technique

The polyester fabric was printed via a sublimation transfer technique using the former silk-screened printed-paper. The heat source is a 40 × 25 cm flat-bed press. Transfer times and temperatures are (30, 60 s) and (170, 190, and 210 °C), respectively. The printed samples are settled to cool at room temperature, before the paper is removed.

##### Color strength (K/S) and CIELAB color parameters

The color strength (K/S), CIELAB color parameters L*, a*, b*, and ∆E of the prints are evaluated by Hunter Lab (Ultra scan-PRO D65, USA, at max of λ_max_680 (nm). The color strengths (K/S) of printed samples were measured using the Kubelka–Munk relationship;

$${\text{K}}/S = {{\left( {1 - {\text{R}}} \right)^{2} } \mathord{\left/ {\vphantom {{\left( {1 - {\text{R}}} \right)^{2} } {2{\text{R}}}}} \right. \kern-0pt} {2{\text{R}}}}$$

where S refers to the scattering coefficient, K refers to the absorbance coefficient and R refers to reflectance. L* specifies the sample brightness, a* specifies the sample red or green shift, b* specifies the sample yellow or blue area shift, and ∆E is a single value that determines the total differences between the sample parameters a*, b*, and L* and the standard color^18^. Fastness measurements of washing, perspiration, and light were assessed according to standard methods AATCC technical manual, method 8 (1989) 68, 23 (1993); 15 (1989) 68, (1993) 30 and 16(2004) respectively^19^. The rubbing fastness was assessed according to AATCC 1993b^10^.

## Results and discussion

### Characterization of the prepared TiO_2_/RHSA pigments

#### XRF analysis of the prepared RH, RHSA and TiO_2_/RHSA pigments

The main constituent of RH, RHSA and TiO_2_/RHSA are represented in Table 2. From the Table it is clear that, titanium dioxide did not exist in rice husk, and it was slightly present in RHSA with percentage 0.02, while in case of TiO_2_/RHSA core–shell pigment, its presence was about 13.25 and this means that it forms more than 10% of the whole compound. This was compensated on the silica percentage that was lowered to 79.36 in case of core–shell pigment, i.e. there is indirect relationship between the concentration of TiO_2_ and SiO_2_. Also it can be concluded from this analysis that, the calculated ash yield from RH raw material was 14.23% and this obtained ash has a faint gray color containing 82.93% silica particles and 11.36% weight loss on ignition (LOI).

**Table 2:** Main constituents for rice husk (RH), rice husk silica ash (RHSA) and TiO_2_/RHSA pigments.

Main constituents (wt.%)	RH	RHSA	TiO_2_/RHSA
SiO_2_	65.43	82.93	79.36
TiO_2_	–	0.02	13.25
Al_2_O_3_	0.10	0.34	0.22
MnO	0.02	0.06	0.04
Fe_2_O_3_^tot^	0.07	0.14	0.13
MgO	0.07	0.52	0.43
CaO	0.29	1.04	0.65
Na_2_O	0.04	0.27	0.11
K_2_O	0.54	2.21	1.23
P_2_O_5_	0.10	0.57	0.50
SO_3_	0.19	0.33	0.12
Cl	0.08	0.22	0.20
L.O. I	33.07	11.35	3.76

#### Morphology of the prepared TiO_2_/RHSA pigments

**Figure 1:**
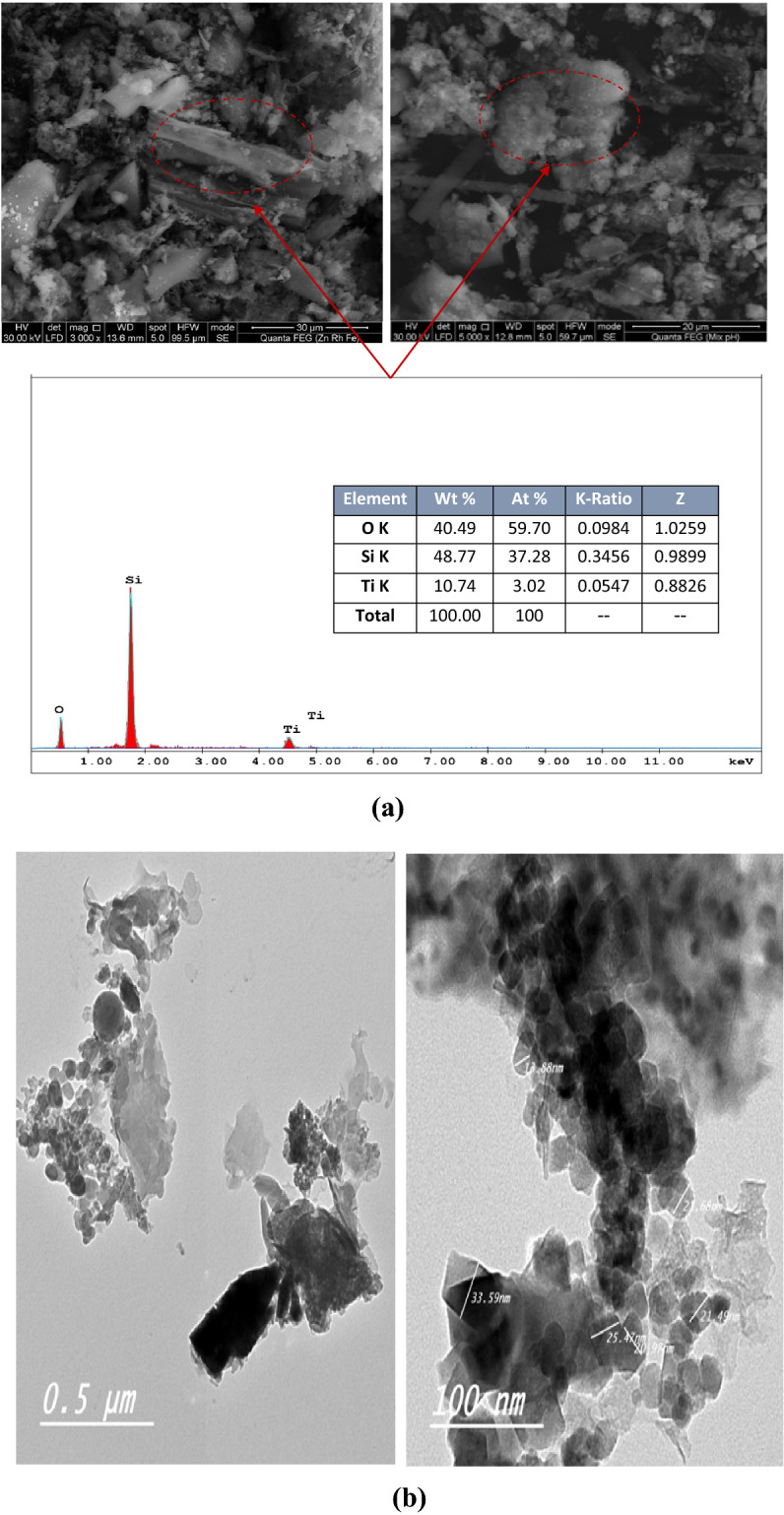
(**a**) SEM/EDX of TiO_2_/RHSA and (**b**) TEM photo of TiO_2_/RHSA.

SEM photos and EDX analysis of TiO_2_/RSHA core–shell pigments shown in Fig. 1a cleared that, the platelet particles of titanium dioxide are overlapping on the spherical-like shapes of silica in addition to the appearance of some rod-like structures, which can be referred to non-complete canlcined RH. This overlapping between the platy structure of titanium dioxide and the spheres of RH silica can provide a good way to enhancing the paper mechanical properties by strengthening its interstitial portions. In addition, EDX analysis, which estimates the elements on the surface up to 1 µ depth, showed the appearance of titanium dioxide in a percentage nearly similar to that found through the XRF analysis (about 13.25%) indicating that almost all the titanium dioxide is located on the surface of silica and traces only are in the bulk which is in good agreement with XRF results.

TEM photos in Fig. 1b, confirmed the appearance of titanium dioxide particles as precise sharp dimensional plates surrounding the spheres of silica that appeared because of the rice husk calcinations, confirming the structure of the new core–shell pigment.

### Properties of hand sheets loaded with prepared fillers

The physical, mechanical and barrier properties of (unloaded, loaded hand sheets with CaCO_3_, TiO_2_, RHSA and TiO_2_/RHSA) composite fillers were summarized as follows.

#### Filler retention, grammage and bulk density

**Figure 2:**
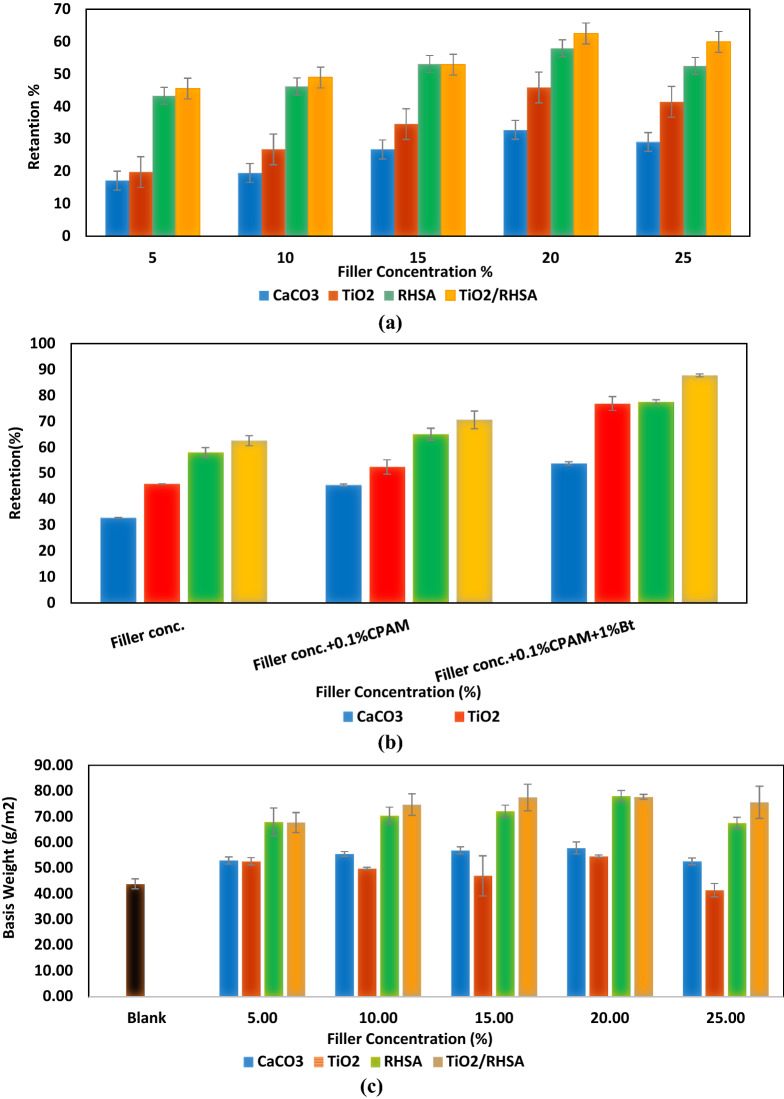

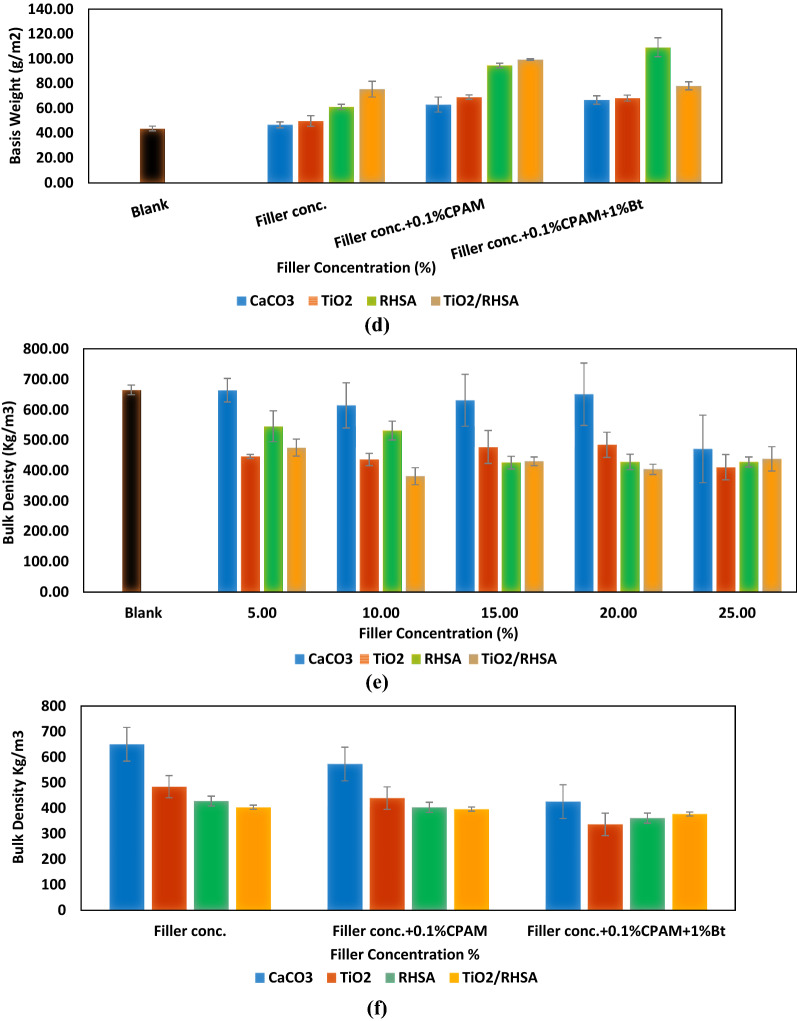
Effect of filler concentration, retention aid addition on retention percent (**a**,**b**), basis weight (**c**,**d**) and bulk density (**e**,**f**) of paper sheets.

The retention of fillers has always been a hot theme in the research area of papermaking, because the retention efficiency strongly influences the cost of the product, cleanliness of the papermaking system, and pollution load of the disposal system. Therefore, it was necessary to study the retention performances of CaCO_3_, TiO_2_, RHSA and TiO_2_/RHSA, to evaluate their effect on the paper sheets made from bleached kraft bagasse. The results in Fig. 2a and b indicated an increase in the retention values with the increase in the filler concentration. The use of CaCO_3_ and TiO_2_ fillers with concentrations (5–25%) only resulted in filler retention of (17.20 ± 2.81–32.79 ± 3.21%) and (19.81 ± 4.29–45.87 ± 4.33%), respectively. Comparing between using CaCO_3_ and TiO_2_ fillers with RHSA and TiO_2_/RHSA, all the pigments offered high filler retention^13^. Nevertheless, RHSA offered the highest retention with increased percentage of 33.83%. Also, the retention values varied from (45.51 ± 2.81%) to (62.47 ± 3.52%) for papers coated with TiO_2_/RHSA core–shell pigments, and the optimum filler concentration that yielded highest filler retention without addition of retention aid was 20% for CaCO_3_, TiO_2_, RHSA and TiO_2_/RHSA.

The higher retention value obtained for RHSA and TiO_2_/RHSA was (57.95 ± 2.35%) and (62.47 ± 3.52%), respectively which is due to the hydrophobicity of silica and its high content. According to the results of these trials, 0.1% cationic polyacrylamide (CPAM) was added to 1% bentonite. The presence of CPAM enhanced CaCO_3_, TiO_2_, RHSA and TiO_2_/RHSA aggregation. Agglomerated fillers are satisfactorily large to retain inside the sheet and fill the gaps in the fibers. The long-chain CPAM adsorption on the surfaces occurred through either bridging or charge neutralization. The results indicated that; (1) retention was extremely low in the absence of additives, (2) addition of CPAM improved CaCO_3_, TiO_2_, RHSA and TiO_2_/RHSA retention results, and (3) retention improvement was greatly affected by adding bentonite. The increase in the retention of fillers as a result of the addition of bentonite with the different flocculants can be attributed to the fact that, it can provide a bridge between flocculent covered fibers and fillers^16,20^. The retentions were (53.75 ± 0.65), (76.87 ± 3.13%), (77.47 ± 1.53%) and (87.68 ± 1.32%) for S9, S10, S11 and S12, respectively. In fact, the presence of ionized functional groups in sufficient amounts is necessary to give the right conformation to the polymer and ensure good level of flocculation/retention (bridging ability). Adsorption of TiO_2_/RHSA core–shell particles screened out repulsive electrostatic interactions between the polymers and the surfaces. The best retention results (87.68 ± 1.32%) were offered by S12.

Accordingly, the basis weight increased with the content of CaCO_3_, TiO_2_, RHSA and TiO_2_/RHSA due to flocculants/bentonite addition. Paper bulk density is defined as the relation between paper thickness and its grammage, and it was found that the presence of filler particles tends to announce more spaces between fibers that otherwise would be tightly bonded together in the sheet. The change in bulk density values of the loaded hand-sheets was consistent with the variation in filler retention results as can be seen in Fig. 2, showing that the paper hand-sheets containing TiO_2_, RHSA and TiO_2_/RHSA exhibited lower bulk density than either unloaded papers (bleached kraft bagasse) or papers loaded with CaCO_3_. The results also showed that, RHSA particles and TiO_2_/RHSA core–shell pigments at higher concentration (15, 20 and 25% addition) exhibited lower bulk densities than unloaded and loaded samples containing CaCO_3_ and TiO_2_ loaded hand-sheets. Whereas, the bulk density percentage was decreased to 17.96% for papers containing TiO_2_/RHSA core–shell pigments. Addition of 0.1% CPAM and 1% bentonite with the loaded fillers decreased the bulk density of modified paper sheets with the added fillers and this contradicts the retention aid results.

#### Mechanical strength properties of hand-sheets

**Figure 3:**
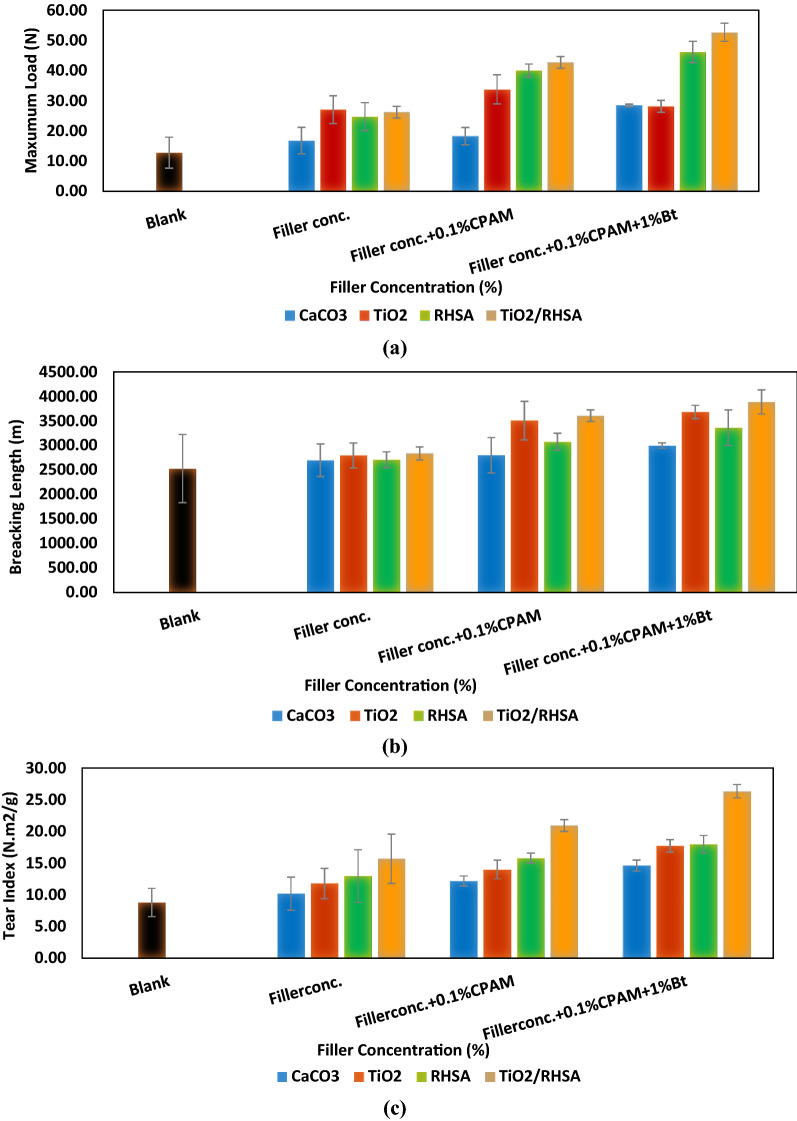

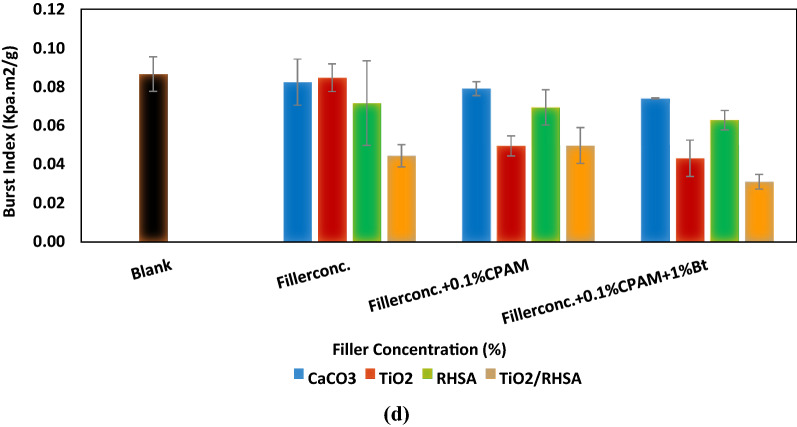
Strength properties for various filler additives in relation with paper composition, (**a**) maximum load, (**b**) breaking length, (**c**) tear index and (**d**) burst index.

Figure 3a–c confirmed that addition of CaCO_3_, TiO_2_, RHSA and TiO_2_/RHSA in the coatings led to a significant increase in the maximum load, breaking length and tear index. Also, adding 0.1% CPAM and 1% Bt to the different pigments offered an improvement in the breaking length that occurred at (53.82%) for S12. This particulate sample offered the highest retention (87.68% ± 1.32) and the smallest particle size. In addition, the same trend was obtained for tear index measurements, whereas the improvement reached (26.34 ± 1.35 N m^2^/g) for S12. Hand-sheets loaded with TiO_2_, RHSA and TiO_2_/RHSA core–shell pigments showed lower burst indices than blank and hand-sheets loaded with CaCO_3_ as can be detected in Fig. 3d. The small particle size of fillers exposed higher relative amount of fiber surface that can be covered by a given mass particle preventing inter-fiber contact over a larger fraction of the available surface area. In addition, TiO_2_/RHSA pigments exerted the lowest hand-sheet burst index, and this can be also attributed to its highest retention value accompanied with the smallest size of silica particles^1,16^.

#### Barrier properties for paper sheets treated with various filler additives

In our study, different barrier properties (air, water vapor, and Cobb value) were evaluated for the loaded paper sheets with CaCO_3_, TiO_2_, RHSA and RHSA/TiO_2_ to compare their effect.

##### Air permeability

**Figure 4:**
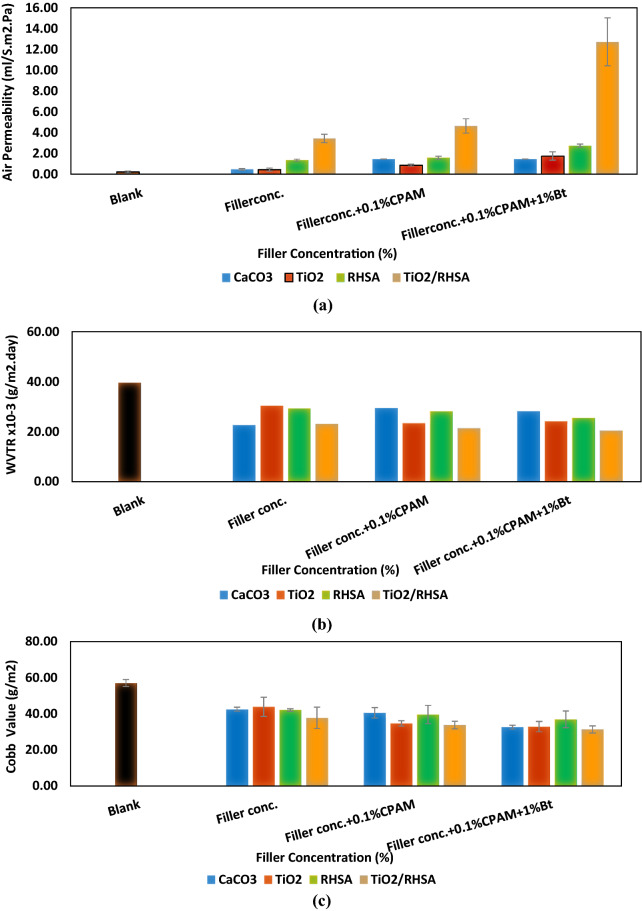
Barrier properties for paper sheets loaded with fillers, (**a**) air permeability, (**b**) water vapor transmittance and (**c**) cobb value.

The porous structure of paper has an important role in paper competence concerning further numerous applications; this porosity is usually correlated to the air resistance that reverses air permeability. Since the level of pulp aggregation influences the porous structure of the paper, air permeability measurements can be a sign of the possible mechanisms of aggregation developed by the various additive systems and their effect on paper quality. Air permeability increased by the application of the different additives significantly. The highest air permeability was achieved for paper filled with the different pigments in the presence of CPAM and bentonite. Air permeability results in Fig. 4a showed an increase by adding additives to the pulp, and the highest increase was in case of paper sheets loaded by S12 to offer air permeability (12.72 ± 2.78 ml/S m^2^ Pa). This indicates that the formed particles can contribute to regulating the porosity of the composite materials. Furthermore, it is considered that filler particles allowed larger spaces (percolation path) between fibers, decreasing their compactness and thereby leading to increased bulkiness of the material^7,20–22^.

##### Water vapor transmission rate (WVTR)

The WVTR of the paper sheet is the volume of water vapor passing through the paper unit area and time under definite conditions. WVTR is measured at steady state, and it can be predicted that the modification of the cellulose fibers decreased the water vapor transmission. WVTR for unloaded and loaded hand-sheets of 50% RH is shown in Fig. 4b, where a decrease in WVTR for the loaded hand-sheets was noticed compared to unloaded ones.

To explain this finding, it is necessary to understand the mechanism of water vapor transmission through cellulose fibers. First, water molecules condense on the surface, then they solubilize into the loaded cellulose fibers and thereafter they can diffuse through it. After that, these molecules leave the surface of the paper to the other side. Generally, it can be noticed that addition of CaCO_3_, TiO_2_, RHSA and TiO_2_/RHSA particles as fillers to paper matrix decreased its WVTR, because of the increase in the particle numbers, which cordially widens the path of water vapor. The results also confirmed that S12 significantly influenced the WVTR of paper sheets. However, the obtained values in case of unloaded sample were (39.60 × 10^−3^ ± 4.40) and then they were reduced to (20.47 × 10^−3^ ± 0.53) (g/m^2^ day) in case of S12 for paper sheets loaded with (TiO_2_/RHSA + 0.1% CPAM + 1% Bt.), showing an improvement of 48.23% compared to unloaded paper sheets. This is because of the presence of RHSA particles, which can create zigzag way for penetrating water vapor via the paper matrix, resulting in a hydrophobicity improvement of paper sheets^23,24^. This helps to avoid printing paste, dye, or ink from penetrating the inner paper structure and settling on the paper surface.

##### Cobb value

The Cobb test is essential to estimate the ability of the paper to resist the penetration of water and the quantity of water that can be absorbed by the surface of paper sheets; it also evaluates the quality of paper over a given time. The Cobb values for different additive systems in relation to paper composition are represented in Fig. 4c; the results of this test confirmed a significant decrease in Cobb values after loading the fillers in the pulp, from (57.03 ± 1.03 g/m^2^) for unloaded paper sheet to (37.77 ± 5.93 g/m^2^) for paper coated with S4. Addition of cationic polyacrylamide and bentonite in the pulp led to a decrease in Cobb values, and hence improved sizing degree of the paper sheets can be clearly detected. It is supposed that CPAM which is used for the retention, can help in attaching CaCO_3_, TiO_2_, RHSA, TiO_2_/RHSA particles on cellulose fibers with a negative charge, increasing the quantity of retention on paper sheets^25^.

From the previous results, the loaded paper sheets with RHSA particles can be used in printing papermaking by reducing the average diameter of the paper pores and this will be expressed in details in the SEM.

### SEM of paper sheets

**Figure 5:**
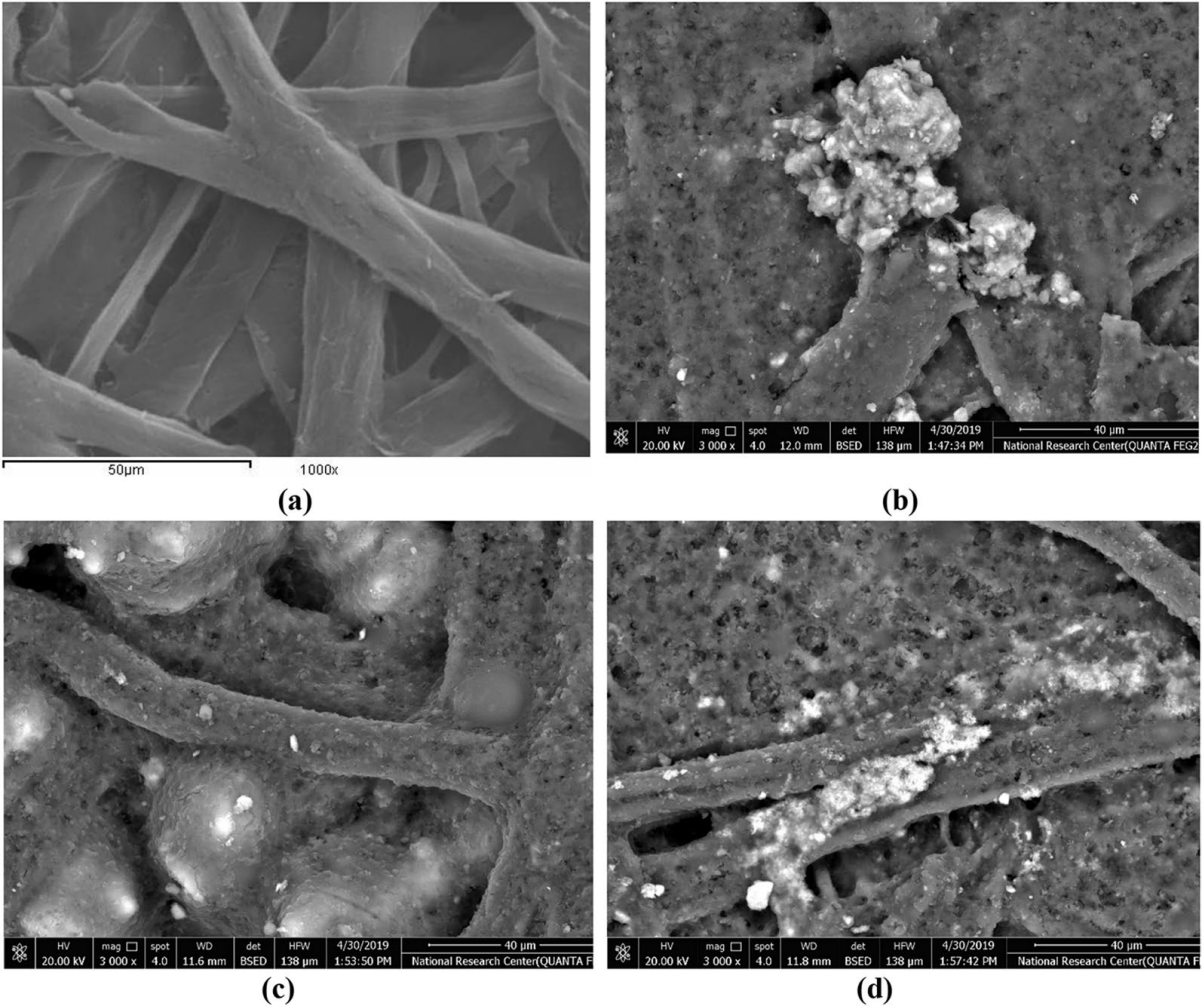
SEM images for paper sheets, (**a**) S0 (blank bagasse), (**b**) S9 (20% CaCO_3_ + 0.1% CPAM + 1% Bt), (**c**) S11 (20% RHSA + 0.1% CPAM + 1% Bt) and (**d**) S12 (20% TiO_2_/RHSA + 0.1% CPMA + 1% Bt).

The surface morphology of paper sheets loaded with CaCO_3_, RHSA, and TiO_2_ was different from that of unmodified bleached kraft bagasse pulp as can be seen in Fig. 5. Almost all the pores were closed by the pigment particles, but a fibrous surface can be still visible. Incorporating fillers in paper sheets led to the agglomeration of deposited particles on the fiber surfaces in fiber-based matrices. Markedly, the core–shell (TiO_2_/RHSA) pigment particles exhibited higher retention, and this was obvious in the SEM images due to their smaller particle size and their high distribution compared to CaCO_3_ and RHSA particles Fig. 5d. Adding 0.1% CPAM and 1% Bentonite with the pigment led to homogenous close in fiber morphology in the paper sheets. In addition, Fig. 5 cleared that the surface encapsulation due to the filler modification was quite evident, which helps in the enhancement of the compatibility with cellulosic pulp fibers that occurred, making the surface smoother and tighter^7,26^. Accordingly, tighter cellulose fiber combinations resulted in higher mechanical strength, which explains the improvements in hand sheet mechanical strength.

### Color analysis of printed papers

**Figure 6:**
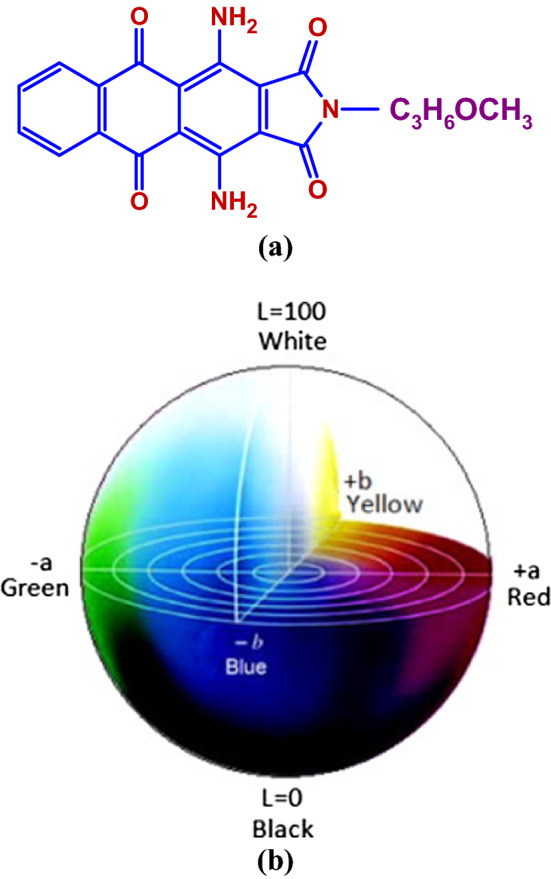
(**a**) Chemical structure of Turquoise Blue S-GL (Disperse Blue 60) and (**b**) The color parameters L*, a* and b* model from CIELAB color space^27^.

The (K/S) values of silk-screen printed papers by Turquoise Blue S-GL dye, Fig. 6a (Disperse Blue 60) are illustrated in Table 3. And as it is well known that, K/S values express color intensity and depth, and it was clear in the study that K/S values were enhanced in most cases than commercial ones. This indicated that, the papers have retained more dye on their surfaces, and samples S9, S11 and S12 offered the highest K/S values. Such K/S values demonstrated that the added fillers improved bagasse paper ability to keep the coated dye on the upper paper layers, while reducing dye absorbency into deeper layers (as demonstrated by SEM micrographs) (Fig. 5). The color parameters are evaluated using the CIELAB system L*, a*, and b* scales and the results are given in Table 3. A three-dimensional model of CIELAB color space is designed according to the opponent-colors theory in Fig. 6b^27^.The values revealed that, K/S values are in indirect relationship with L* values, confirming that the color became darker and vice versa. In addition, the ∆E of printed samples showed notable changes that strongly confirmed the presence of a color difference between printed samples. Maximum color difference values were observed for printed-paper sheet samples S12, S11 followed by S9. Moreover, it is important to note that from a* and b* values, all samples showed a blue shift.

**Table 3:** The color strengths (K/S) and CIELAB color parameters of printed papers using silk screen technique.

Sample	K/S	L*	a*	b*	∆E
Commercial transfer paper (CTP)	6.53	36.21	3.26	14.66	49.89
S0	7.69	34.21	2.60	13.72	51.63
S1	6.77	34.94	2.25	12.00	50.73
S2	7.38	34.03	1.88	12.50	51.68
S3	7.71	32.98	2.46	11.81	52.68
S4	7.35	34.51	2.88	13.63	51.33
S9	7.72	32.65	2.73	12.27	53.04
S10	7.37	34.38	3.02	13.95	51.50
S11	8.52	31.58	2.76	12.65	54.15
S12	9.01	28.87	2.43	10.26	56.69

### Thermo-gravimetric (TGA) analysis

Thermo-gravimetric (TGA) analysis is a very valuable technique to study the thermal behavior of specific materials^28^. It is important to evaluate the effect of added fillers on the thermal stability performance of printed-paper upon exposure to the high temperature press in the transfer process.

**Figure 7:**
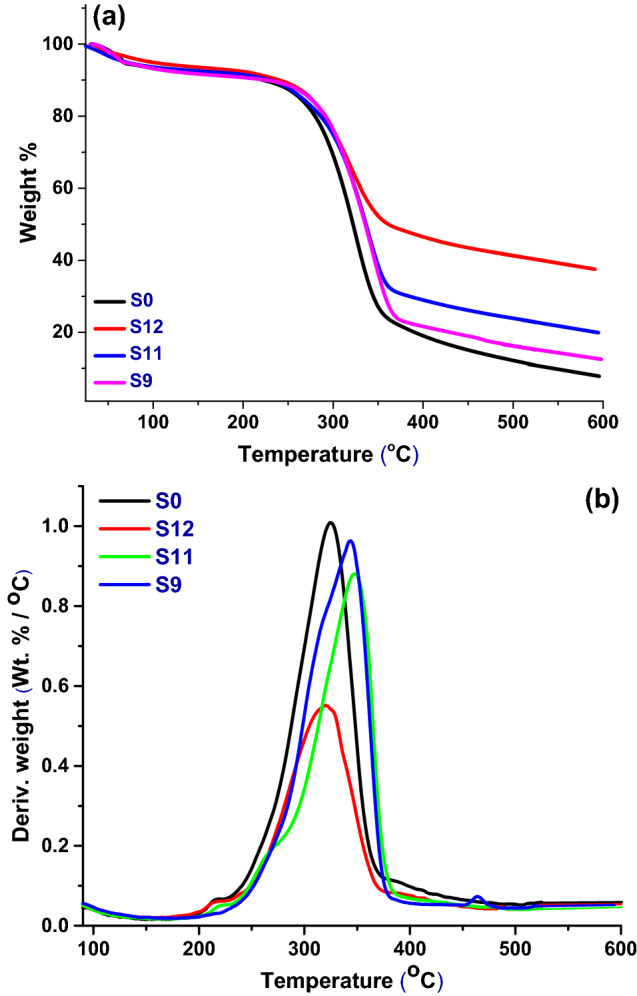
TGA (**a**) and DTG (**b**) profiles of printed papers, S0 (blank bagasse), S9 (20% CaCO_3_ + 0.1% CPAM + 1% Bt), S11 (20% RHSA + 0.1 % CPAM + 1 % Bt) and S12 (20% TiO_2_/RHSA + 0.1 % CPMA + 1% Bt).

TGA and DTG profiles of unloaded and some selected modified paper sheets under an inert atmosphere was done, the selection depended on K/S printing results. The results of TGA and DTG data are summarized in Table 4 and shown in Fig. 7. It is clear that, all paper samples showed one main stage of weight loss apart from the primary weight loss in the range of 30–80 °C, which was attributed to the evaporation of absorbed water. In addition, it was observed that all modified samples showed higher thermal stability and char percentage formation than unloaded paper sheets. Comparing these modified samples to unloaded bagasse paper sheets, modified paper sample S12 containing (20% RHSA/TiO_2_ + 0.1% CPAM + 1% Bt) offered enhancement in its thermal stability, followed by S11 (20% RHSA + 0.1% CPAM + 1% Bt) then finally S9 containing (20% CaCO_3_ + 0.1% CPAM + 1% Bt).

**Table 4:** TGA & DTG data of blank and modified printed papers.

Paper sample	T_10%_ (°C)	*T*_*max*_ (°C)	*T*_*f*_ (°C)	CR_500°C_ (wt.%)
S0	222.2	325	373	12.22
S9	225.53	344	384	16.3
S11	230	337	378	23.91
S12	240.35	319	375	41.28

Where T_10%_ is the temperature where the sample losses 10% from its weight during the process, T_max_ is the temperature corresponding to major decomposition process, *T*_*f*_ is the temperature were the decomposition process is finished and CR_500°C_ (wt.%) is the Char residue percent at temperature corresponding to constant weight loss.

Unloaded bagasse paper exhibited less thermal stability with lower ability to char formation than all other tested paper samples as shown in Table 4, due to cellulose decomposition which occurred via a depolymerization mechanism of glycosyl units, resulting in the formation of high-boiling products (HBP) (levoglucosan). Those HBPs under higher temperatures might decompose into lighter flammable gases. In addition, it is well known that the presence of additives, pigments, fillers or metals can markedly affect the thermal stability, decomposition pathway and decomposition products^7,13,29^.

The char residue at 500 °C (CR500 °C) increased from 12.22% for unloaded papers to 41.28% for S12 and 23.19% for S11, then 16.36% for S9. The higher char value was associated with S12 which is related to the presence of both silica ash and TiO_2_, exhibiting a double effect on structure stabilization. The presence of ash in S11 also stabilized its structure, while presence of calcium carbonate improved the thermal stability of bagasse paper, but not as much as in cases of TiO_2_ and RHSA. Those enhanced effects attributed to the ability of added fillers to stabilize the structure of bagasse and direct the thermal decomposition to less volatile and non-flammable decomposition pathways. In addition, the added fillers catalyzed the dehydration decomposition mechanism of bagasse paper, favoring the formation of carbonaceous char (formation of CO_2_, CO, H_2_O and solid char) and minimizing the depolymerization of cellulose to levoglucosan^30^. Furthermore, S12 exhibited flame retardant properties through the reduction in decomposition temperature (319 °C compared to 325 °C for blank bagasse), and increasing in char yield formation (41.28% compared to 12.22% of blank bagasse). This was done by reducing levoglucosan formation and catalyzing both the decomposition and dehydration reactions. The results revealed that the added fillers aided the stabilization and thermal stability of the bagasse structure. The printing and thermo-gravimetric results of the modified papers revealed that they have the potential to be used as heat transfer printing papers for polyester sublimation printing.

### Heat transfer printing of polyester fabrics using silkscreen printed modified heat transfer papers

In sublimation transfer printing, cellulose paper is considered as the first dye carrier candidate for dispersed dyes. Since it has no affinity for these dyes, high quality, uniform-structure paper with good mechanical and thermal behavior was required. Therefore, the structural properties of cellulose paper sheets were modified, in order to be utilized in transfer printing applications. Inorganic fillers are frequently used to provide paper characteristics, including opacity, bulk, and smoothness, that are necessary for paper quality^31,32^.

Silica is characterized for its high surface area, hydrophilicity, chemical stability, and inert nature, as well as durability and compatibility. For that, silica is considered as an excellent paper filler material. Nevertheless, due to its porous nature, the ink can diffuse along the coating layer; therefore its structure must be adjusted to regulate the penetration of printing paste into the paper structure in order to fulfil the needs of heat transfer application. Larger particles and a relatively high surface area are generated by the TiO_2_ located as a shell on the surface of the porous silica core^33^.

Furthermore, titanium can minimize coating cracking, and enhance the thermal stability at higher temperatures. Treatment of paper sheets with such a core–shell structure may reduce paste penetration into the inner paper structure and limit water absorption by imparting the necessary level of hydrophobicity and leaving the dye film attached to the surface paper. Moreover, TiO_2_ can considerably improve the thermal conductivity of paper surfaces, preventing the mottled effect brought on by the paper varied thermal insulation, while in contact with the heating element as well as producing a homogenous and uniform image transfer. TiO_2_/RHSA core–shell treatments may reduce the affinity of dye vapor for paper fibers, coatings, and thickening agents used in printing paste. This can improve the affinity of the dye for the fibers and may lead to a significant amount of ink or dye being released from the paper surface onto fabrics^31–35^.

In sublimation heat transfer printing, dispersed dye that was printed on paper turns from the solid state to the gaseous state without even going through the liquid state (sublimation) under certain conditions. At higher temperatures, the molecules of synthetic fabric are opened to allow the dye vapor to go inside. After cooling, the polyester molecules are settled and absorbed over the entire dye. This is interesting because the dye is absorbed inside the fabric, unlike other printing processes, where the color is set on the surface only.

**Figure 8:**
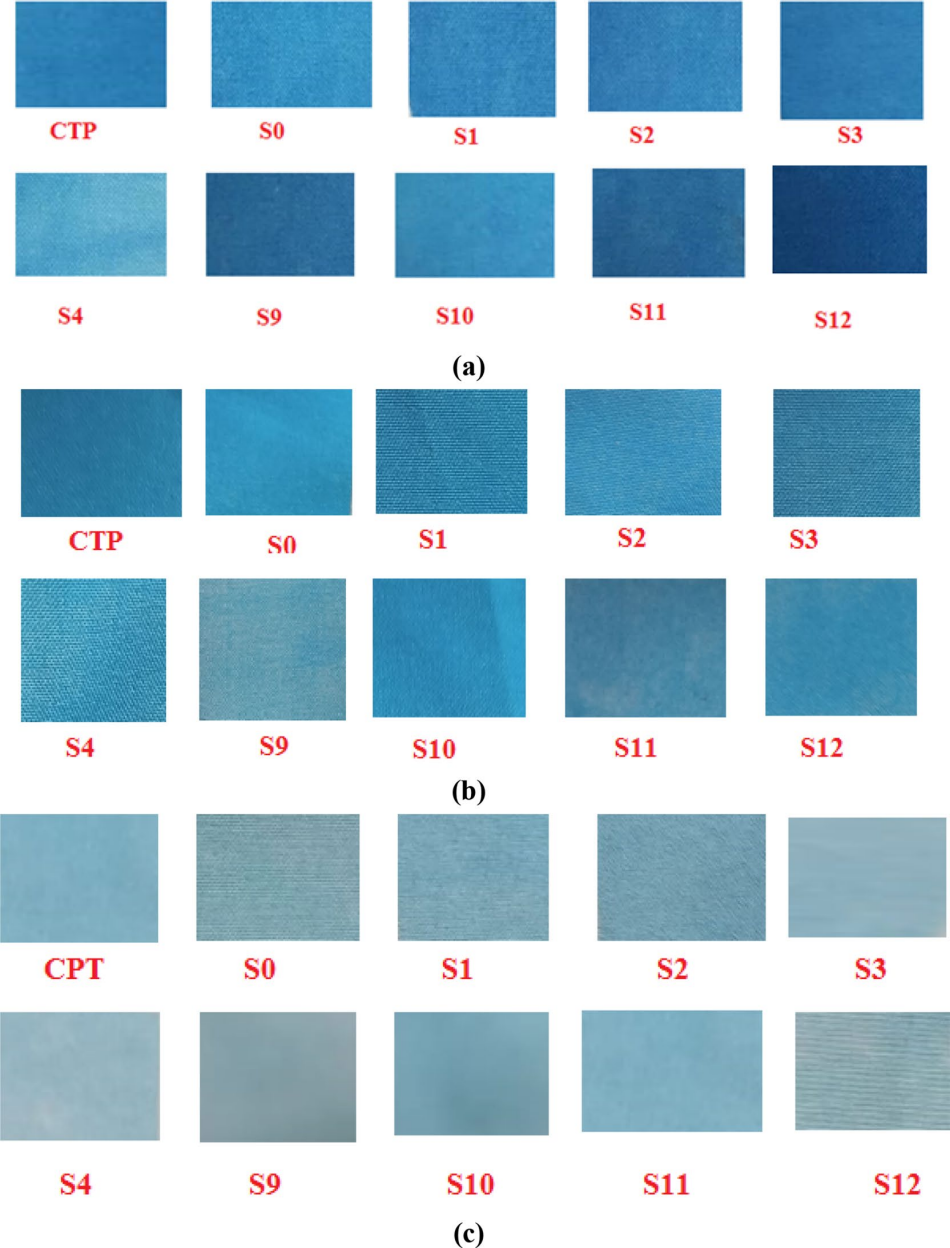
Photographic image of heat transfer-printed polyester fabrics at (**a**) 3%, (**b**) 2% and (**c**) 1% dye shade.

In order to evaluate the homogeneity and color uniformity of the printed image generated from modified and tested paper sheets, three printing runs with different dye concentrations have been performed. Figure 8a–c illustrate the photographic images of polyester fabric printed via selected modified paper sheets at 1, 2, and 3% dye concentration, respectively.

The images demonstrated that all of the studied printed samples by treated paper had sharp, regular edges and exhibited high levels of homogeneity and regularity in the colors that were transferred onto the fabrics, which may indicate the homogeneity and regularity of the materials loaded on the surface of the paper.

Color differences are evaluated by ∆E; if its numerical value is higher than 5, a variation in color will be visible to the naked eye; if it is increased to higher value than 12, an absolute difference in color will be observed. Table 5 compares each sample's ∆E value against itself to illustrate the degree of color difference. The results revealed that all samples offered acceptable color leveling and homogeneous color dispersion, especially at low dye shades^36^.

**Table 5:** ∆E value of heat transfer printed polyester fabrics at different dye shade.

Sample	∆E		
	1%	2%	3%
Commercial transfer paper (CTP)	2.23	0.46	9.8
S0	1.95	1.68	7.89
S1	0.36	0.32	8.81
S2	0.45	2.41	8.26
S3	2.7	1.70	1.25
S4	2.25	6.01	2.46
S9	0.98	6.11	2.08
S10	3.00	0.26	8.4
S11	0.98	7.04	2.06
S12	0.45	5.2	2.42

The modified silk-screen printed papers were used as heat transfer printing papers in the transfer printing of polyester fabrics. The ability of the printed papers to uniform dye-release under different transfer conditions of temperature and time was evaluated. In Table 6, K/S results of heat transfer printed polyester fabrics using commercial and modified screen printed papers at different times and temperatures were given. The results estimated that, K/S of the polyester increased with increasing the time and temperature of the transfer process, indicating that the dye release is in direct relationship with time and temperature. The highest K/S values were obtained with printed polyester fabrics at 210 °C for 60 s, and the maximum color release was comparable to that of commercial transport paper S9, S11 and S12. This may be due to that the modifications in these samples have enhanced the dye-release in a smooth and uniform manner from their surfaces, leading to homogenous dye capture by polyester fabrics.

**Table 6:** The K/S of transfer printed polyester fabrics using different modified screen-printed papers at different transfer conditions.

Time (s)	Temperature (°C)					
	170		190		210	
	30	60	30	60	30	60
Commercial transfer paper (CTP)	0.24	0.55	0.98	2.80	4.67	11.36
S0	0.14	0.27	0.38	1.92	2.48	4.36
S1	2.49	7.49	2.49	7.49	2.49	7.49
S2	0.15	0.55	0.40	1.79	2.51	7.6
S3	0.16	0.33	0.60	2.01	3.33	8.81
S4	0.19	0.38	0.75	2.90	3.86	9.79
S9	0.22	0.47	0.82	3.07	4.26	11.01
S10	0.18	0.35	0.67	2.30	4.04	9.72
S11	0.22	0.46	0.98	2.82	4.54	11.02
S12	0.24	0.49	0.94	2.98	4.82	11.02

Table 7 showed the color parameters as well as the light, washing, and perspiration fastness values at 210 °C and 60 s. According to the results, all printed polyester fabrics showed a blue shift. Furthermore, ∆E for all printing samples ranged between 34 and 45, indicating that there were successful color transfers between the papers and fabrics. The greater the color difference (∆E) value, the more color gained and hence more intense color can be observed^37^.

**Table 7:** The colour parameters and fastness properties of transfer printed polyester fabrics using modified screen-printed papers at 210 °C and 60 s.

Samples	L*	a*	b*	∆E	Rubbing fastness		Light	Washing fastness			Perspiration					
											Acidic			Alkaline		
								St		Alt	St		Alt	St		Alt
					Wet	Dry		Cotton	Wool		Cotton	Wool		cotton	wool	
Commercial transfer paper(CTP)	56.76	− 18.09	− 38.0	46.2	4–5	4–5	5–6	4–5	4–5	4–5	4–5	4–5	4–5	4–5	4–5	4–5
S0	58.22	− 17.93	− 36.4	44.2	4–5	4–5	5–6	4–5	4–5	4–5	4–5	4–5	4–5	4–5	4–5	4–5
S1	57.04	− 18.11	− 37.8	45.9	4–5	4–5	5–6	4–5	4–5	4–5	4–5	4–5	4–5	4–5	4–5	4–5
S2	56.86	− 17.54	− 37.7	45.7	4–5	4–5	5–6	4–5	4–5	4–5	4–5	4–5	4–5	4–5	4–5	4–5
S3	58.44	− 18.32	− 36.6	44.3	4–5	4–5	5–6	4–5	4–5	4–5	4–5	4–5	4–5	4–5	4–5	4–5
S4	62.04	− 18.79	− 34.5	41.0	4–5	4–5	5–6	4–5	4–5	4–5	4–5	4–5	4–5	4–5	4–5	4–5
S9	66.77	− 18.07	− 29.6	34.9	4–5	4–5	5–6	4–5	4–5	4–5	4–5	4–5	4–5	4–5	4–5	4–5
S10	56.48	− 17.48	− 37.9	46.0	4–5	4–5	5–6	4–5	4–5	4–5	4–5	4–5	4–5	4–5	4–5	4–5
S11	61.77	− 18.22	− 33.7	40.4	4–5	4–5	5–6	4–5	4–5	4–5	4–5	4–5	4–5	4–5	4–5	4–5
S12	60.64	− 18.52	− 35.0	42.0	4–5	4–5	5–6	4–5	4–5	4–5	4–5	4–5	4–5	4–5	4–5	4–5

**Figure 9:**
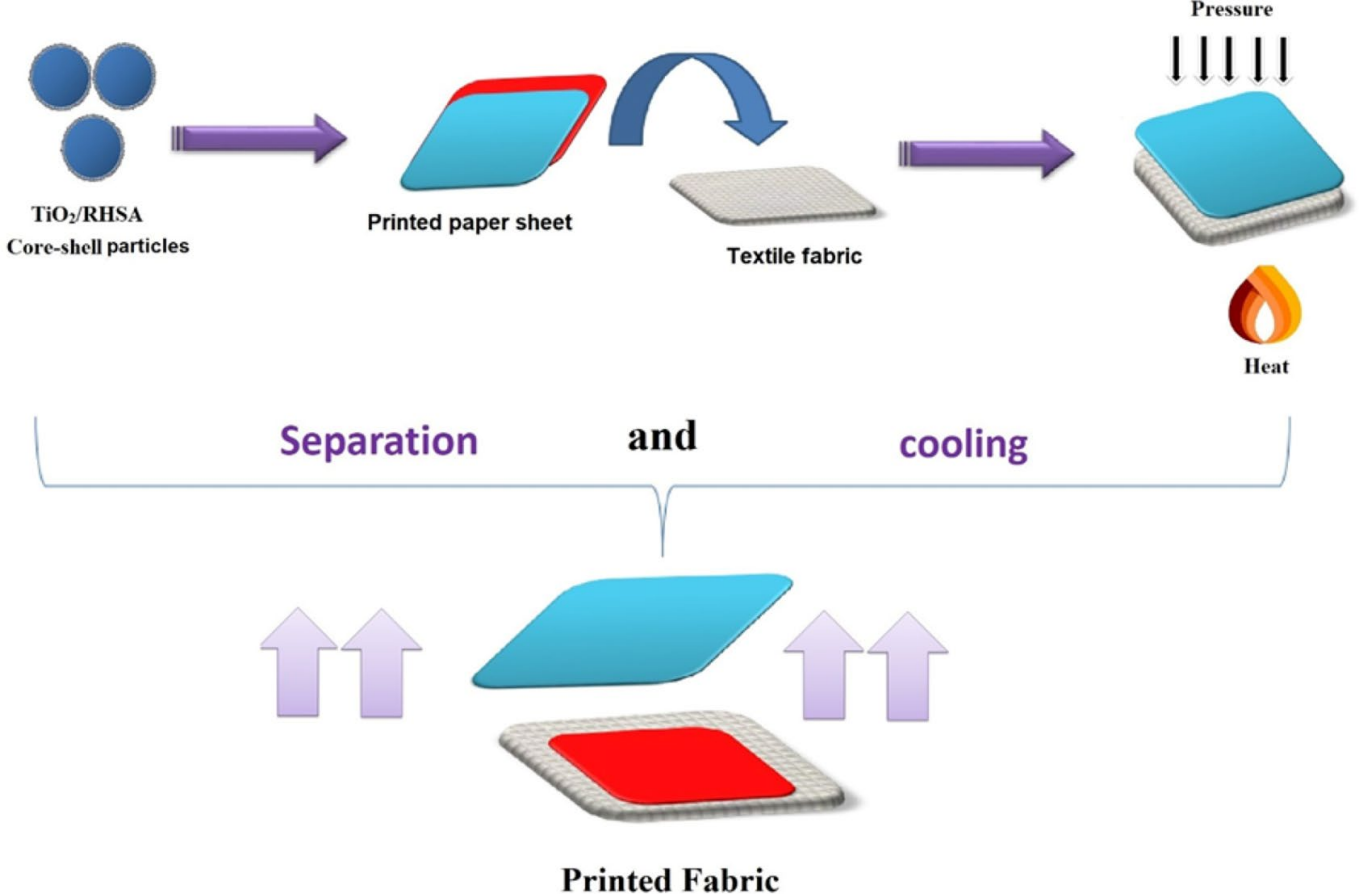
Graphical representation of heat transfer printing papers.

In terms of printed fabric fastness, all printed samples offered excellent stability against washing, perspiration, wet and dry rubbing, and light. Because of this finding, it is now possible to conclude that the modified bagasse papers can provide a shorter transfer time, lower cost, and machine use without cockling, cracking or burning allowing them to be used as high-release transfer printing papers in the transfer printing of polyester fabrics. Graphical representation of heat transfer printing papers presented in Fig. 9.

## Conclusions

In this work, a new core–shell pigment (TiO_2_/RHSA) based on silica ash waste covered with thin layer of titanium dioxide was loaded on paper sheets in the presence of CPAM and bentonite. These additions were done to improve drainage and filler retention in paper hand sheets.

The core–shell TiO_2_/RHSA pigment was prepared at 750 °C and it contains low content of TiO_2_ as a shell covering the RHSA, but in spite of its low concentration, it causes an enhancement of the different mechanical and barrier properties.

The surface morphology of modified paper sheets indicated that incorporating pigments in the paper sheets led to the agglomeration of deposited particles on the fiber surfaces in the fiber based matrices. In addition, paper sheets coated with TiO_2_/RHSA were more homogenous than those treated with CaCO_3_ and RHSA. The results obtained for hand sheets loaded by TiO_2_/RHSA showed an improvement in breaking length and tear index at (53.82%) and (26.34 ± 1.35 N m^2^/g), respectively with a lowering in the burst index. The network created by CPAM, bentonite (Bt) blended with (TiO_2_/RHSA) pigments on paper sheet substrate is highly dense and organized. This resulted in the creation of smooth distribution of particles and strong surface that fills the pores in the paper matrices and increased the air, decreased water vapor transmission, and cobb value of the samples modified with (TiO_2_/RHSA) pigment compared to the blank (unloaded paper). The results showed that, modified silkscreen printed papers could be used as sublimation heat transfer printing papers in polyester fabrics successfully.

## Data Availability

All data generated or analyzed during this study are included in this published article.
